# Fermentability of a Novel Galacto-Oligosaccharide Mixture by *Lactobacillus* spp. and *Bifidobacterium* spp.

**DOI:** 10.3390/molecules23123352

**Published:** 2018-12-18

**Authors:** Suwapat Kittibunchakul, Thomas Maischberger, Konrad J. Domig, Wolfgang Kneifel, Hoang-Minh Nguyen, Dietmar Haltrich, Thu-Ha Nguyen

**Affiliations:** 1 Food Biotechnology Laboratory, Department of Food Science and Technology, BOKU—University of Natural Resources and Life Sciences Vienna, A-1190 Vienna, Austria; suwapatkt@gmail.com (S.K.); thomas.maischberger@gmx.at (T.M.); hoangminh02sh@gmail.com (H.-M.N.); dietmar.haltrich@boku.ac.at (D.H.); 2 Food Microbiology and Hygiene Laboratory, Department of Food Science and Technology, BOKU-University of Natural Resources and Life Sciences Vienna, A-1190 Vienna, Austria; konrad.domig@boku.ac.at; 3 Food Quality Assurance Laboratory, Department of Food Science and Technology, BOKU-University of Natural Resources and Life Sciences Vienna, A-1190 Vienna, Austria; wolfgang.kneifel@boku.ac.at; 4 Department of Biotechnology, The University of Danang-University of Science and Technology, Nguyen Luong Bang 54, 550000 Danang, Vietnam

**Keywords:** prebiotics, β-galactosidase, galacto-oligosaccharides, *Lactobacillus*, *Bifidobacterium*

## Abstract

This study aimed to investigate the specific growth stimulation of certain desired intestinal bacteria by a novel galacto-oligosaccharide mixture, which was produced with a β-galactosidase from a potential probiotic *Lactobacillus* isolate that contained mainly oligosaccharides of β-1,3 and β-1,6 glycosidic linkages (termed Lb-GOS) using single-strain fermentations. The composition of this Lb-GOS mixture was 33.5% disaccharides, 60.5% trisaccharides, 4.8% tetrasaccharides, and 1.0% pentasaccharides with a negligible amount of monosaccharides, lactose, and lactobionic acid (0.3%). Eight *Lactobacillus* spp. strains and three *Bifidobacterium* spp. strains were used in single-strain fermentations to determine the fermentation activity scores of this Lb-GOS preparation compared to two commercially available prebiotic mixtures, 4′GOS-P and Vivinal GOS (V-GOS). The highest scores were obtained when *L. reuteri* Lb46 and the two *Bifidobacterium* strains, *B. animalis* subsp. *lactis* Bif1 and Bif3, were grown on these galacto-oligosaccharide mixtures. In addition, the Lb-GOS mixture was found to have higher fermentation activity scores; hence, it stimulated the growth of these probiotic strains more than 4′GOS-P and V-GOS, which may be attributed to the different glycosidic linkage types that are found in the Lb-GOS mixture compared to the other two commercial preparations. These findings suggested that the Lb-GOS mixture that is described in this work should be of interest for the formulations of new carbohydrate-based functional food ingredients.

## 1. Introduction

The human colonic microbiota is composed of more than 1000 different species [1]. Most of these species are bacteria, some of which have been related to the health and well-being of the host [2]. Among the beneficial gut bacteria, bifidobacteria and lactobacilli are numerically predominant, and are most frequently considered for health-promoting effects [3]. The concept of prebiotics was first introduced by Gibson and Roberfroid [4], was later revised by them [5,6], and has been recently updated by Bindels et al. [7]. According to a recent definition of the prebiotic concept, a prebiotic is “a non-digestible compound that, through its metabolization by microorganisms in the gut, modulates the composition and/or activity of the gut microbiota, thus conferring a beneficial physiological effect on the host” [7]. Prebiotic oligosaccharides can serve as fermentable substrates for certain members of the gut microorganisms, and have been found to selectively stimulate beneficial gut flora such as bifidobacteria and lactobacilli, as well as inhibit “undesirable” bacteria such as pathogens [8,9]. At present, commercially important prebiotic oligosaccharides are available mainly in the Japanese, European, and United States (USA) markets, and include fructo-oligosaccharides (FOS), galacto-oligosaccharides (GOS), and lactulose. Candidate prebiotics include lactosucrose, arabinoxylans, glucans, resistance starch, soybean oligosaccharides, isomalto-oligosaccharides, xylo-oligosaccharides, and gentio-oligosaccharides [10]. In addition, the polysaccharide inulin is frequently used because of its prebiotic effect. Based on the criteria of (i) resistance to mammalian digestive enzymes, (ii) the selective fermentation by intestinal microflora, and (iii) the stimulation of growth and/or activity of intestinal bacteria associated with health and well-being, only inulin/FOS, GOS, and lactulose are fulfilling these requirements for prebiotics as documented in several studies, although promise exists for several other dietary oligosaccharides [5,6,11]. 

Galacto-oligosaccharides (GOS), or transgalacto-oligosaccharides as they are sometimes called, can be produced by the transgalactosylation of lactose catalyzed by β-galactosidases. Transglycosylation becomes an important reaction competing with hydrolysis under certain conditions such as high lactose concentration, as well as the presence of saccharide moieties as acceptors of the galactosyl–enzyme complex, and depends strongly on the enzyme source that is employed. In addition, the temperature and the composition of the reaction mixture are key factors affecting the rate of transgalactosylation, lactose solubility, and enzyme operational stability [12,13]. Functional effects of GOS on human health have been summarized [12,14]. The concept of developing prebiotics for specific probiotic strains has been proposed [15]. To this end, proven probiotic isolates are selected for their ability to generate certain oligosaccharides, either by directly using the microorganism or specific enzymes derived thereof. These oligosaccharides might then be preferentially utilized by the producing probiotics, as was shown for GOS produced by *Bifidobacterium* spp. derived β-galactosidases [16,17,18]. 

A preliminary investigation of the prebiotic potential of oligosaccharides can be made using *in vitro* cultures of intestinal bacteria [19]. We were interested in evaluating the fermentation activity of an oligosaccharide mixture based on the fermentation profiles of single-strain cultivations on these sugars. We investigated and compared the fermentation activity of a novel galacto-oligosaccharide mixture (Lb-GOS), produced by β-galactosidase from a potential probiotic *Lactobacillus* isolate, with two commercial GOS products (4′GOS-P, β1→4 linked galacto-oligosaccharides from Yakult Honsha, Tokyo, Japan and Vivinal, termed V-GOS, from Borculo Domo Ingredients, Zwolle, the Netherlands) using single-strain fermentations. 

## 2. Results

### 2.1. Preparation of Galacto-Oligosaccharide Mixture

**Figure 1:**
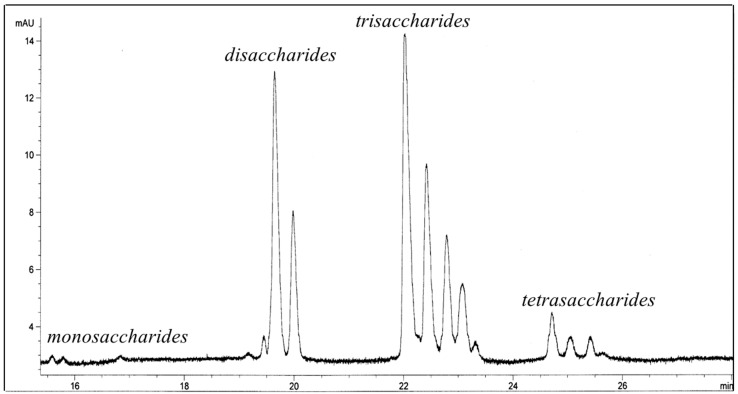
Separation of galacto-oligosaccharide mixture (Lb-GOS) after removal of monosaccharides and lactose using capillary electrophoresis (CE).

The GOS mixture obtained after lactose conversion contained 48% (*w*/*w*) monosaccharides, 26.5% (*w*/*w*) lactose, 9.8% (*w*/*w*) non-lactose disaccharides, 14.7% (*w*/*w*) trisaccharides, and 1.0% (*w*/*w*) tetrasaccharides, as analyzed by high-performance anion exchange chromatography with pulsed amperometric detection (HPAEC-PAD). The final purified Lb-GOS product was of very high purity containing 99.1% GOS, 0.6% lactobionic acid, 0.1% d-glucose, 0.1% d-galactose and 0.1% lactose (Table 1, Figure 1). β-galactosidase from this *Lactobacillus* sp. has a preference for the formation of β-(1→3) and β-(1→6) bonds in its transgalactosylation mode, and hence the main GOS products were identified to be β-d-Gal*p*-(1→6)-d-Glc (allolactose), β-d-Gal*p*-(1→3)-d-Gal, β-d-Gal*p*-(1→6)-d-Gal, β-d-Gal*p*-(1→3)-d-Glc, β-d-Gal*p*-(1→3)-β-d-Gal*p*-(1→4)-d-Glc (3′-galactosyl lactose), and β-d-Gal*p*-(1→6)-β-d-Gal*p*-(1→4)-d-Glc (6′-galactosyl lactose). Compared to the commercial GOS mixtures, the Lb-GOS product produced by a lactobacillal β-galactosidase differs not only in composition, but also in the size and linkage types of its components. Lb-GOS contains disaccharides and trisaccharides with β(1→6) and β(1→3) linkages as its main components, whereas 4′GOS-P and V-GOS were reported to consist mainly of β(1→4) linked galacto-oligosaccharides [20,21]. This pure Lb-GOS mixture was used for further studies on the fermentation activity of GOS.

### 2.2. Effects of Various Galacto-oligosaccharides as the Main Carbohydrate Substrates on the Single-Strain Fermentations

Three strains of bifidobacteria, eight strains of lactobacilli, *E. faecium* En61, *E. coli* DSM 613, *S. epidermis* DSM 20044, *K. oxytoca* DSM 6673, and *C. freundii* DSM 30039 were grown on glucose (0.5% *w*/*v*), various oligosaccharides (0.5% *w*/*v*), and on their basal complex media without an added sugar. The optical densities of the cultures (OD_600_) were measured during the course of these cultivations, with the maximum values presented in Table 2. The *Lactobacillus* strains that were tested can be divided into two groups based on their ability to grow on galacto-oligosaccharides, glucose, and the blank medium. A group of five strains, *L. reuteri* Lb46, *L. reuteri* Lb21, *L. acidophilus* Lb19, *L. acidophilus* Lb71, and *L. acidophilus* Lb105, showed similar growth characteristics with good growth on all of the test substrates, while growth on the blank was low. These strains showed slightly better growth on glucose and Lb-GOS than that on the commercial GOS products (Table 2); in some instances, growth was even better on the oligosaccharide mixture than on glucose. Three other *Lactobacillus* strains, *L. rhamnosus* Lb29, *L. paracasei* subsp. *paracasei* Lb16, and *L. paracasei* subsp. *paracasei* Lb20, showed very good growth on glucose; in fact, it was the best growth on glucose out of all of the strains that were tested. However, they showed only moderately good growth on Lb-GOS and V-GOS, and slow growth on 4′GOS-P compared to that of the *Lactobacillus* strains in the first group. These three strains also showed the highest cell densities on carbohydrate-free basal media among the *Lactobacillus* strains tested. The three strains of *Bifidobacterium* showed comparable growth characteristics, with moderate to good growth on glucose and Lb-GOS, slow growth on V-GOS, and relatively poor growth on 4′GOS-P. *E. faecium* En61 showed the highest growth on Lb-GOS compared to the other test substrates. The strains of *E. coli* DSM 613, *S. epidermis* DSM 20044, *K. oxytoca* DSM 6673, and *C. freundii* DSM 30039 were grouped as ‘enteric bacteria’ in this study, and showed moderate growth when there was no C-source present in the medium, and slow growth in the presence of GOS.

**Figure 2:**
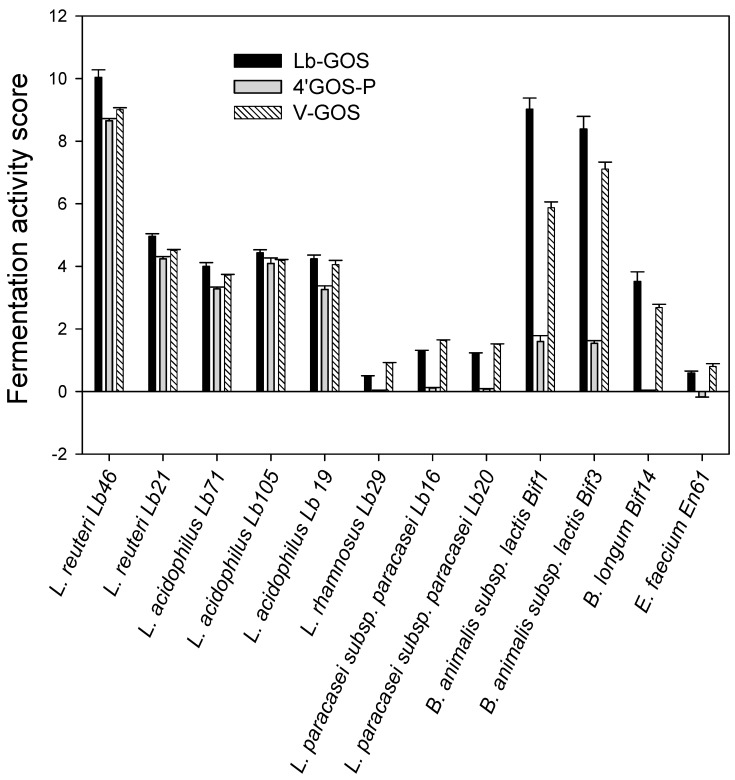
Fermentation activity scores (FAS), calculated by using Equation (1), for various strains of *Bifidobacterium* and *Lactobacillus* grown on different prebiotic galacto-oligosaccharide mixtures at 37 °C and for 24 h. Lb-GOS, galacto-oligosaccharides produced by β-galactosidase from *Lactobacillus* sp.; 4′GOS-P, β1→4 linked galacto-oligosaccharides; V-GOS, Vivinal galacto-oligosaccharides.

The maximum OD_600_ values displayed in Table 2 were used for the calculation of the fermentation activity scores (Figure 2) using Equation (1). The highest scores were for *L. reuteri* Lb46 and *B. animalis* subsp. *lactis* Bif1 grown on Lb-GOS (10.04 and 9.02, respectively), and for *L. reuteri* Lb46 paired with V-GOS and 4′GOS-P (9.00 and 8.65, respectively), followed by *B. animalis* subsp. *lactis* Bif3 grown on Lb-GOS and V-GOS (8.39 and 7.11, respectively). Low scores were found for the group of *L. rhamnosus* Lb29, *L. paracasei* subsp. *paracasei* Lb20, and *L. paracasei* subsp. *paracasei* Lb16, as well as for *E. faecium*, especially when grown on 4′GOS-P. In addition, the three *Bifidobacterium* strains grown on 4′GOS-P showed consistently lower fermentation activity scores compared with V-GOS and the novel Lb-GOS mixture. 

## 3. Discussion

Prebiotics, which commonly are oligosaccharides, are metabolized selectively in the gastrointestinal tract by beneficial bacteria associated with health and well-being. These carbohydrates can thus positively modulate the colonic microbiota, which exerts an important influence on host health [5,8,9,22]. Different methods such as pure culture fermentations of single, selected strains [23,24,25,26] and *in vitro* fermentations of mixed bacterial populations, particularly fecal bacteria [19,27,28], have been used as preliminary screening tools for prebiotic activities, whereas *in vivo* fermentations of non-digestible carbohydrates in animals and human subjects can be used for evaluating the prebiotic effects of different oligosaccharide mixtures [5,29]. Pure culture fermentations are performed in appropriate basal media supplemented with the respective prebiotics, and the increase in cell numbers is quantified by the turbidimetry of the cultures or by viable cell count. A better model for investigating the interactions between the gut populations is pH-controlled batch cultures. Here, fermentation is again based on basal media, with the test carbohydrate being the sole fermentable substrate present, but the use of fecal bacterial populations allows for an investigation of the interactions, competition, and cross-feeding during growth on the selected substrate. Changes in the concentration of intestinal bacteria in feces are monitored using molecular techniques such as fluorescence in situ hybridization (FISH) or real-time PCR [25,30]. Alternatively, *in vitro* colonic models and the ^13^C labeling of substrates can be used to study the prebiotic activity [31].

Comparative studies on different oligosaccharides are still limited. Results between studies are sometimes difficult to compare [32], and no overall conclusion concerning the prebiotic efficiency of different oligosaccharides or structure/function relationships have yet been found [32,33]. In a comparative *in vitro* study by Watson et al. [34], FOS, GOS, and lactulose were oligosaccharides with growth-promoting effects against lactobacilli and bifidobacteria, in which GOS possessed a superior potential to the majority of the observed strains over FOS or inulin. In another study, GOS, isomalto-oligosaccharides, lactulose, and FOS were found to be metabolized well by all of the tested bifidobacterial strains with varying growth rates, while the efficient utilization of xylo-oligosaccharides was limited to some strains e.g., *B. lactis* [35]. Bouhnik et al. [36] tested the capacity of different oligosaccharides to stimulate fecal bifidobacteria in a placebo-controlled *in vivo* study; FOS, GOS, soybean oligosaccharides, and type III resistant starch were found to be bifidogenic. The ability of oligosaccharide uptake generally seems to vary within the genus of *Lactobacillus* and *Bifidobacterium*; hence, different growth rates on various oligosaccharides can be observed. In a recent study, the growth of single strains of *Bifidobacterium*, *Lactobacillus*, and *Streptococcus* on various trisaccharides (including 4′-galactosyl-lactose, 6′-galactosyl-lactose, 4′-galactosyl-lactulose, and 6′-galactosyl-lactulose) was evaluated, and in general, these strains grew faster on the trisaccharides with a β(1→6)-galactosyl moiety [37]. According to Thongaram, et al. [38], the GOS-utilizing capacity varying among bifidobacteria and lactobacilli relied on the degree of polymerization of GOS, and was strain-dependent as well. However, the preferential fermentation of short-chain oligosaccharides by bifidobacteria was reported [39].

The strains of *Bifidobacterium* and *Lactobacillus* as well as *E. faecium* in this study were selected, since some isolates are already established as probiotic strains and are used in food/feed products, or they have potentially probiotic properties such as a positive impact on the establishment and balance of the normal microflora, protection from gastrointestinal diseases, the production of important digestive enzymes, the alleviation of symptoms of lactose intolerance, cholesterol-lowering effects, stimulation of the immune system, and managing inflammatory bowel disease [40,41]. When comparing the GOS mixtures in single-strain cultivations, the highest fermentation activity scores were found for the three *Bifidobacterium* strains tested and *L. reuteri* Lb46 grown on purified Lb-GOS. Significantly lower fermentation activity scores were obtained for these *Bifidobacterium* strains when paired with V-GOS, which contains significant amounts of glucose and galactose that can support growth, and especially 4′GOS-P. Presumably, the different glycosidic linkages of the oligosaccharides in the GOS mixtures influence the selective ability of probiotic bacteria to metabolize these carbohydrates, as it is also evident from the maximal optical density values obtained for lactobacilli and bifidobacteria, which are consequently higher when using Lb-GOS compared to 4′GOS-P. An explanation for the above-mentioned observation could be that β-galactosidases from bifidobacteria possess a preference for hydrolyzing β(1→6) and β(1→3) linkages; hence, their growth was stimulated better with Lb-GOS, containing mainly β(1→6) and β(1→3) linked galacto-oligosaccharides, rather than with 4′GOS-P or V-GOS, which are mainly β(1→4) linked galacto-oligosaccharides. 

A large variation in the fermentation activity scores was found in the single-strain fermentations for the different strains of *Lactobacillus*. Interestingly, even different isolates within a single species, such as *L. reuteri* Lb46 and *L. reuteri* Lb21, exhibited significantly different fermentation activity scores. *L. rhamnosus* and *L. paracasei* subsp. *paracasei*, which are phylogenetically closely related [42], showed low fermentation activity scores on all three GOS mixtures. Especially *L. rhamnosus* Lb29 seems unable to utilize GOS, as growth on these substrates was only comparable to the blank on the complex basal medium without an added carbohydrate. It was discussed previously that these differences may be due to diversity among lactobacilli and the presence of genes coding for the metabolic systems that are necessary for the transportation and utilization of a particular prebiotic as a carbon source [10,24]. Among the substrates tested with *Lactobacillus* spp., the novel Lb-GOS mixture, which was produced with an enzyme from *Lactobacillus* sp., was the best-performing substrate, giving the highest fermentation activity scores and optical density values for all of the lactobacilli tested (Figure 2, Table 2). This is obvious, especially when comparing its growth and fermentation activity scores with 4′GOS-P, which is also free of monosaccharides. These results substantiate the idea that oligosaccharides produced with enzymes from probiotic strains can have a more pronounced effect on the growth of these probiotic strains [16,17,18,43]. 

## 4. Materials and Methods

### 4.1. Chemicals

All of the chemicals were purchased from Sigma (St. Louis, MO, USA) or Oxoid (Basingstoke, Hampshire, UK), and were of the highest quality available, unless otherwise stated. Glucose oxidase (GOD) from *Aspergillus niger* (lyophilized, 205 U/mg enzyme preparation) was from Fluka (Buchs, Switzerland) and horseradish peroxidase (POD) (lyophilized, 210 U/mg) was from Boehringer (Mannheim, Germany). The test kit for the determination of d-galactose/lactose was from Megazyme (Bray, Ireland).

### 4.2. Strains and Culture Conditions

Three *Bifidobacterium* strains (*B. animalis* subsp. *lactis* Bf1, *B. animalis* subsp. *lactis* Bf3, and *B. longum* Bf14), eight *Lactobacillus* strains (*L. paracasei* subsp. paracasei Lb16, *L. acidophilus* Lb19, *L. paracasei* subsp. paracasei Lb20, *L. reuteri* Lb21, *L. rhamnosus* Lb29, *L. reuteri* Lb46, *L. acidophilus* Lb71, and *L. acidophilus* Lb105) and *Enterococcus faecium* En61 were obtained from the culture collection of the Food Microbiology Laboratory, BOKU-University of Natural Resources and Life Sciences Vienna. The strains *Escherichia coli* DSM 613, *Staphylococcus epidermis* DSM 20044, *Klebsiella oxytoca* DSM 6673, and *Citrobacter freundii* DSM 30039 were obtained from DSMZ (Deutsche Sammlung von Mikroorganismen und Zellkulturen GmbH, Braunschweig, Germany). *Bifidobacterium* spp. were maintained in brain–heart infusion (BHI) broth medium (beef heart and calf brain 17.5 g/L, Na_2_HPO_4_·2H_2_O 2.5 g/L, peptone 10 g/L, NaCl 5 g/L, l-cysteine·HCl 0.5 g/L) supplemented with 1% (*w*/*v*) glucose and 15% (*w*/*v*) glycerol at −72 °C. *Lactobacillus* spp. were maintained at −72 °C in De Man, Rogosa, and Sharpe (MRS) broth (peptone 10 g/L, di-potassium hydrogen phosphate 2 g/L, meat extract 8 g/L, di-ammonium hydrogen citrate 2 g/L, yeast extract 4 g/L, sodium acetate 5 g/L, magnesium sulfate 0.2 g/L, Tween 80 1 g/L, manganese sulfate 0.04 g/L) containing 2% (*w*/*v*) lactose and 15% (*w*/*v*) glycerol. *E. faecium* was maintained in *Corynebacterium* broth (peptone 10 g/L, yeast extract 5 g/L, NaCl 5 g/L) supplemented with 0.5% (*w*/*v*) glucose and 15% (*w*/*v*) glycerol. *E. coli*, *K. oxytoca*, and *C. freundii* were stored at –72°C in nutrient broth (peptone 5 g/L, meat extract 3 g/L) with 15% (*w*/*v*) glycerol; *S. epidermis* was maintained in supplemented *Corynebacterium* broth, as described above. These latter four strains are grouped under the term ‘enteric bacteria’, which describes the autochthonous enteric strains of relevance for the gut microbiota in this study.

### 4.3. Prebiotic Oligosaccharide Mixtures

The commercial prebiotic galacto-oligosaccharide mixture Vivinal (V-GOS), containing 40% monosaccharides and lactose and 60% oligosaccharides, was obtained from Borculo Domo Ingredients (Zwolle, the Netherlands). The mixture of 4′GOS-P from Yakult Honsha (Tokyo, Japan) is a purified product with a purity of 99.9% of galacto-oligosaccharides, which are mainly β(1→4) linked oligosaccharides. Lb-GOS, containing mainly β(1→3) and β(1→6) linked oligosaccharides, was produced and purified using β-galactosidase from *Lactobacillus* sp. Discontinuous GOS production was carried out at 23 °C using purified β-galactosidase (five U/mL) in a five-liter scale stirred tank reactor (total volume of six liters) and 206.5 g/L lactose dissolved in 50 mM of sodium phosphate buffer (pH 6.0) containing 2 mM of MgCl_2_. When the desired degree of lactose conversion of 73% was reached, the reaction mixture was heated to 98 °C to inactivate β-galactosidase and clarified by centrifugation. To remove non-converted lactose from the sugar mixture, the enzymatic conversion of lactose to lactobionic acid was carried out as described previously using the enzyme cellobiose dehydrogenase (CDH) from *Sclerotium rolfsii* [44]. After the enzymatic oxidation step, the GOS solution was centrifuged and filtered to remove insoluble material, and then applied onto two ion-exchange chromatography columns in series using a strong cation exchange resin, Lewatit^®^ S2528 (Bayer AG, Leverkusen, Germany), and a medium basic anion exchange resin, Lewatit^®^ S4328 (Bayer AG, Leverkusen) for the removal of ions as described previously [45]. For the separation of the GOS from d-glucose and d-galactose, the strong acidic cation exchange material Unibead UBK-530 (Mitsubishi Chemical Industries, Tokyo, Japan) was used. 

### 4.4. Enzyme Activity Assay and Protein Measurement

The determination of β-galactosidase activity was carried out at 30 °C using 22 mM of *o*-nitrophenyl-β-d-galactopyranoside (*o*NPG) in 50 mM of sodium phosphate buffer (pH 6.5) as substrate, as previously described [46]. The reaction was initiated by adding 20 µL of enzyme solution to 480 µL of the substrate solution, and then incubated for 10 min using an Eppendorf thermomixer compact (Eppendorf, Hamburg, Germany) with an agitation of 600 rpm. The reaction was stopped by adding 750 µL of 0.4 M of Na_2_CO_3_. The release of *o*-nitrophenol (*o*NP) was measured by determining the absorbance at 420 nm. One unit of *o*NPG activity was defined as the amount of enzyme releasing one µmol of *o*NP per minute under the described conditions. Protein was determined by the method of Bradford [47] with the BioRad Coomassie Blue reagent (Marnes-la-Coquette, France) using bovine serum albumin as the standard.

### 4.5. Sugar Analysis

Monosaccharide analysis. d-Glucose was measured enzymatically by the coupled GOD/POD assay, as described previously [20]. For the determination of d-galactose, the lactose/d-galactose test kit from Megazyme was used. 

Oligosaccharide analysis. Capillary electrophoresis (CE) and high-performance anion exchange chromatography with pulsed amperometric detection (HPAEC-PAD) (Dionex, Sunnyvale, CA, USA) were used for the qualitative and quantitative analysis of galacto-oligosaccharides. A capillary electrophoresis system with a UV-DAD detector (Agilent Technologies, Palo Alto, CA, USA) together with a fused silica capillary (internal diameter of 25 µm) equipped with a bubble cell detection window (bubble factor of five) was used for carbohydrate analysis. Carbohydrate samples were derivatized with 2-amino pyridine for CE analysis, as given in detail in [20]. HPAEC-PAD analysis was carried out on a Dionex DX-500 system consisting of a GP50 gradient pump (Dionex), an ED 40 electrochemical detector with a gold working electrode (Dionex), and an Ag/AgCl reference electrode (Dionex). Separations were performed at room temperature on a CarboPac PA-1 column (4 × 250 mm) connected to a CarboPac PA-1 guard column (Dionex).

### 4.6. Single Strain Cultivations and Automated Turbidimetry

Frozen cultures of bifidobacteria and lactobacilli were activated by streaking onto BHI agar containing one g/L of glucose, or MRS agar supplemented with 2% (*w*/*v*) lactose, respectively, and incubating anaerobically at 37 °C for 24–48 h as required. *E. coli*, *K. oxytoca,* and *C. freundii* were streaked onto nutrient agar, and *S. epidermis* and *E. faecium* were streaked onto *Corynebacterium* medium containing 0.5% (*w*/*v*) glucose. These five strains were incubated aerobically at 37 °C for 24 h. After the given incubation times, one single colony from each plate was picked and transferred to an appropriate medium to obtain pure cultures. Each strain was then transferred from the agar plates into appropriate liquid media (without a carbohydrate source), and a series of dilutions was prepared to obtain the inoculum for further experiments. The respective inocula (100 µL) and 200 µL of the appropriate fresh medium (containing the C-source) were placed in each micro-plate well (Honeycomb 2 plates; Labsystem, Les Ulis, France). The final carbohydrate concentration (glucose or prebiotic oligosaccharides) in these media was 0.5% (*w*/*v*), and the starting optical densities (OD_600_) were approximately 0.001. For the cultivation of bifidobacteria and lactobacilli, anaerobic conditions were maintained by adding the enzyme system Oxyrase^®^ (Oxyrase Inc., Mansfield, OH, USA) to a final concentration of 2% (*v/v*), and the reading plates were kept airtight. The inoculated honeycomb plates were then placed in the reading chamber of a Bioscreen C MBR (Labsystems, Vantaa, Finland) and incubated at 37 °C. The cultures were mixed for 30 s before each reading was taken by the setting ‘medium-intensity shaking mode’. The optical densities (OD_600_) of the cultures were measured with readings being taken every 30 min for 24 h. Growth of the cultures in the media without an added carbohydrate source was also monitored as a blank. All of the measurements were carried out in triplicate.

### 4.7. Fermentation Activity Score

Fermentation activity scores (*FAS*), i.e., a measure for the ability of one selected strain to utilize a sugar mixture for growth in comparison with other enteric bacteria, were determined using the following Equation (1):

(1) $$FAS=\frac{\Delta Pro_{prebiotic}}{\Delta Pro_{blank}}-\frac{\Delta Ent_{prebiotic}}{\Delta Ent_{blank}}$$

$$\Delta Pro_{prebiotic}=Pro_{prebiotic}^{\max}-Pro_{prebiotic}^{\min}$$

$$\Delta Ent_{prebiotic}=Ent_{prebiotic}^{\max}-Ent_{prebiotic}^{\min}$$

$$\Delta Pro_{blank}=Pro_{blank}^{\max}-Pro_{blank}^{\min}$$

$$\Delta Ent_{blank}=Ent_{blank}^{\max}-Ent_{blank}^{\min}$$

$Pro_{prebiotic}^{\max}$ and $Ent_{prebiotic}^{\max}$ are the highest optical densities OD_600_ obtained during 24 h of the growth of probiotic bacteria and enteric bacteria, respectively, on prebiotic oligosaccharides; $Pro_{prebiotic}^{\min}$ and $Ent_{prebiotic}^{\min}$ are the lowest optical densities OD_600_ obtained at the inoculation of probiotic bacteria and enteric bacteria, respectively, when using prebiotic oligosaccharides; $Pro_{blank}^{\max}$ and $Ent_{blank}^{\max}$ are the highest optical densities OD_600_ obtained during 24 h of growth of probiotic bacteria and enteric bacteria, respectively, in the medium without added sugar; and $Pro_{blank}^{\min}$ and $Ent_{blank}^{\min}$ are the lowest optical densities OD_600_ obtained at the inoculation of probiotic bacteria and enteric bacteria, respectively, for the medium without added sugar.

This equation assumes that an increase in the number of probiotic bacteria, bifidobacteria, and lactobacilli gives a positive effect, whilst an increase in the number of enteric bacteria, which in this study are *E. coli* DSM 613, *S. epidermis* DSM 20044, *K. oxytoca* DSM 6673, and *C. freundii* DSM 30039, gives a negative effect. Based on this equation, substrates with a high fermentation activity score support the growth of bifidobacteria, lactobacilli, and *E. faecium*, with the culture optical densities significantly higher than the cultivations on the complex medium without the added carbohydrate.

### 4.8. Statistical Analysis

All of the experiments and measurements were performed at least in triplicate, and the data are given as the mean ± standard deviation when appropriate. The data were analysed using SPSS (SPSS Inc. Chicago, IL, USA; Version 11.0.0). 

## 5. Conclusions

The fermentation activity scores, as determined from single-strain fermentations on several galacto-oligosaccharide substrates, reflect the potential of a given carbohydrate to promote the selective growth of a specific microorganism. This approach of pairing a specific strain with a prebiotic seems especially valid when searching for synbiotic mixtures, in which a certain probiotic strain is paired with a prebiotic supporting its growth. Hence, this highly potential prebiotic GOS mixture described in this work should be of considerable interest for the formulations of new carbohydrate-based functional food ingredients. 

## Figures and Tables

**Table 1:** Composition of the novel Lb-GOS mixture produced using β-galactosidase from *Lactobacillus* sp. after removal of monosaccharides and lactose.

	Composition (% *w*/*w*)
Monosaccharides	<0.2
Lactose	<0.1
Disaccharides	33.5
Trisaccharides	60.5
Tetrasaccharides	4.8
Higher saccharides	<1.0

**Table 2:** Maximum optical densities OD_600_^a^ of single-culture fermentations using different galacto-oligosaccharide mixtures or glucose as the main carbohydrate substrates.

Strain	Carbohydrate Source ^b^				
	V-GOS	4′GOS-P	Lb-GOS	Glucose	Blank ^c^
*L. reuteri* Lb46	1.26 ± 0.03	1.25 ± 0.01	1.38 ± 0.01	1.28 ± 0.04	0.25 ± 0.00
*L. reuteri* Lb21	1.19 ± 0.02	1.19 ± 0.02	1.31 ± 0.01	1.30 ± 0.04	0.34 ± 0.02
*L. acidophilus* Lb19	1.35 ± 0.04	1.19 ± 0.02	1.47 ± 0.01	1.43 ± 0.03	0.40 ± 0.04
*L. acidophilus* Lb71	1.41 ± 0.03	1.36 ± 0.06	1.54 ± 0.01	1.47 ± 0.03	0.43 ± 0.00
*L. acidophilus* Lb105	1.36 ± 0.04	1.39 ± 0.05	1.47 ± 0.05	1.44 ± 0.06	0.38 ± 0.00
*L. rhamnosus* Lb29	1.01 ± 0.01	0.56 ±0.03	0.74 ±0.01	1.64 ± 0.01	0.80 ± 0.03
*L. paracasei* subsp. *paracasei* Lb16	1.33 ± 0.01	0.55 ± 0.01	1.21 ± 0.01	1.55 ± 0.01	0.71 ± 0.10
*L. paracasei* subsp. *paracasei* Lb20	1.33 ± 0.01	0.55 ± 0.02	1.21 ± 0.01	1.54 ± 0.00	0.71 ± 0.02
*B. animalis* subsp. *lactis* Bif1	0.77 ± 0.03	0.34 ± 0.04	0.95 ± 0.03	1.22 ± 0.02	0.25 ± 0.02
*B. animalis* subsp. *lactis* Bif3	0.84 ± 0.04	0.35 ± 0.02	0.95 ± 0.03	1.05 ± 0.02	0.24 ± 0.10
*B. longum* Bif14	0.69 ± 0.06	0.28 ± 0.02	0.79 ± 0.03	0.78 ± 0.03	0.31 ± 0.01
*E. faecium* En61	0.72 ± 0.07	0.46 ± 0.01	0.88 ± 0.10	0.66 ± 0.01	0.67 ± 0.19
*E. coli* DSM 613	0.40 ± 0.01	0.69 ± 0.05	0.38 ± 0.01	0.41 ± 0.02	0.95 ± 0.04
*K. oxytoca* DSM 6673	0.35 ± 0.00	0.45 ± 0.06	0.33 ± 0.05	0.36 ± 0.04	1.01 ± 0.10
*C. freundii* DSM 30039	0.51 ± 0.02	0.73 ± 0.01	0.34 ± 0.04	0.42 ± 0.09	0.89 ± 0.04
*S. epidermis* DSM 20044	0.93 ± 0.02	0.75 ± 0.08	0.88 ± 0.04	0.96 ± 0.02	0.66 ± 0.09

^a^ Maximum values of optical densities OD_600_ obtained during the growth of these strains on different carbohydrate substrates at 37 °C and 24 h. Values given are the mean of three independent experiments ± standard deviation. ^b^ The concentration of the C-source was 0.5% *w*/*v*. V-GOS, Vivinal galacto-oligosaccharides; 4′GOS-P, β1→4 linked galacto-oligosaccharides; GOS, galacto-oligosaccharides produced by β-galactosidase from *Lactobacillus* sp. ^c^ A blank shows growth on the respective complex basal medium without an added sugar.
